# Comparative Analysis of Codon Usage Bias in Transcriptomes of Eight Species of Formicidae

**DOI:** 10.3390/genes16070749

**Published:** 2025-06-27

**Authors:** Wenhui Zhu, Jiawei Wang, Jing Wang, Linlin Nie

**Affiliations:** College of Life Sciences, Shaanxi Normal University, Xi’an 710119, China; ssd2726@snnu.edu.cn (J.W.); wj.3522@snnu.edu.cn (J.W.); alin_0923@snnu.edu.cn (L.N.)

**Keywords:** transcriptomes, codon usage bias, ants, natural selection

## Abstract

**Background:**Ants are among the most widely distributed eusocial insects, and desert ants, in particular, serve as important model organisms for studying animal navigation. **Methods**: In this study, we provide high-quality de novo transcriptomes for eight ant species: *Cataglyphis aenescens* (Nylander, 1849), *Formica approximans* Wheeler, 1933, *Lasius coloratus* Santschi, 1937, *Proformica mongolica* (Emery, 1901), *Proformica muusensis* Zhu, Wu, Duan & Xu, 2022, *Tapinoma geei* Wheeler, 1927, *Tapinoma rectinotum* Wheeler, 1927, and *Tetramorium tsushimae* Emery, 1925. **Results**: The GC content of coding sequences (CDSs) ranged from 43.61% to 46.20%, indicating a slightly AT-rich composition. Codon usage analysis identified 27 to 33 optimal codons per species, the majority of which ended with A or U. **Conclusions**: These transcriptomic resources provide critical insights into codon usage bias and establish a foundation for future research on molecular evolution, gene regulation, and environmental adaptation in ants inhabiting fragile desert ecosystems.

## 1. Introduction

Synonymous codons correspond to the same amino acid but are utilized with varying frequencies across the genome. This uneven usage, termed codon usage bias, arises from multiple evolutionary factors such as mutation pressure, selection on translation efficiency, genetic drift, and biased gene conversion favoring GC content [1,2,3,4]. Several indices, such as the Codon Adaptation Index (CAI) [5], Relative Synonymous Codon Usage (RSCU) [6], and GC content at the third codon position (GC3) [1], are commonly used to quantify codon bias [7,8,9,10,11]. Understanding codon usage patterns is crucial for elucidating the molecular mechanisms underlying genome evolution and gene regulation. To fully appreciate the impact of codon usage bias, it is necessary to explore its functional and evolutionary implications across diverse biological systems [12,13,14,15,16].

Codon usage influences multiple aspects of gene expression, including translation efficiency, tRNA availability, mRNA stability, and protein folding [17,18,19,20,21]. Patterns of codon usage bias vary markedly across species. For instance, dipterans such as *Drosophila* often favor GC-ending codons, likely driven by tRNA abundance and translational efficiency, whereas hymenopterans tend to prefer AT-rich codons, reflecting their genomic base composition and comparatively weaker selection pressure [22,23,24,25,26]. Such biases can constrain evolutionary processes by reducing the rate of synonymous substitutions even under neutral conditions [27]. Given its widespread and complex nature across diverse taxa [28], codon usage bias has become an increasingly important subject of study, particularly with the rapid development of high-throughput sequencing technologies [29,30]. Large-scale genomic and transcriptomic data provide valuable opportunities to better understand the evolutionary forces and translational selection pressures that shape codon usage bias.

Ants (Formicidae) represent the most widespread and species-rich group of eusocial insects, playing critical roles in terrestrial ecosystems. With over 12,000 described species and many more yet to be identified [31], ants offer a valuable model for investigating evolutionary and ecological questions. In particular, desert ants have evolved a suite of physiological and behavioral adaptations that enable them to survive extreme environmental conditions. Some desert-dwelling ants, such as those in the genus *Cataglyphis*, are remarkable for their ability to forage during the hottest parts of the day, often scavenging animals that have perished under extreme desert temperatures [32,33]. Although transcriptomic resources have increased substantially for many desert ants, they remain limited for certain groups, such as the genus *Proformica*.

In this study, we focused on desert ants inhabiting a region that presents a natural gradient of environmental conditions shaped by long-term ecological restoration efforts. The Mu Us Desert, located in northern China, is one of the country’s most ecologically fragile sandy regions. Since the 1990s, large-scale restoration projects have substantially increased vegetation cover, potentially altering the habitats and physiological challenges faced by native insects. This setting offers a unique opportunity to study the effects of environmental change on genome evolution.

In the present study, we generated transcriptomes for eight desert ant species representing six genera (Formicidae): *C. aenescens* (Nylander, 1849) [34], *F. approximans* Wheeler, 1933 [35], *L. coloratus* Santschi, 1937 [36], *P. mongolica* (Emery, 1901) [37], *P. muusensis* Zhu, Wu, Duan & Xu, 2022 [38], *T. geei* Wheeler, 1927 [39], *T. rectinotum* Wheeler, 1927 [39], and *T. tsushimae* Emery, 1925 [40]. Transcriptomic resources for *P. muusensis* are reported here for the first time. These newly generated data provide a valuable foundation for investigating codon usage patterns in ants across different environmental contexts.

RNA-seq enables efficient generation of transcriptomic datasets that are enriched for coding sequences but may also include non-coding and repetitive elements [41,42,43,44]. Leveraging these resources, this study conducts the first comparative analysis of codon usage bias across desert ant species. By examining ants from both natural and restored habitats, we aim to elucidate how environmental conditions and adaptive pressures influence genome evolution and gene expression in Hymenoptera. Our findings not only expand genomic resources for an ecologically important group but also shed light on how ecological restoration may shape the molecular evolution of native insect populations.

## 2. Methods

### 2.1. Sample Collection and RNA Extraction

For de novo transcriptome sequencing, we collected eight Formicidae species representing six genera in the Mu Us Desert. Six of them are native to the Mu Us desert. The other two species (*C. aenescens* and *T. tsushimae*) colonized this environment after artificial planting or aerial sowing of herbs and shrubs. Specimens were initially preserved overnight at 4 °C in RNASafer Reagent (Magen, Shanghai, China) and subsequently stored at −80 °C until RNA extraction. Muscle tissues were used for RNA isolation. For each species, RNA was extracted from pooled samples of four to seven adult individuals. Total RNA was extracted using TRIzol reagent (Invitrogen, Carlsbad, CA, USA) according to the manufacturer’s protocol. RNA integrity and quality were assessed with a NanoDrop spectrophotometer (Thermo Fisher Scientific, Lake Barrington, IL, USA) and an Agilent 2100 Bioanalyzer (Agilent Technologies, Palo Alto, CA, USA). Only samples with an RNA integrity number (RIN) ≥ 8 were used for constructing high-throughput sequencing libraries. The raw sequencing data have been deposited in the National Genomics Data Center Genome Sequence Archive (CRA) under accession number CRA024798.

### 2.2. mRNA-Seq Library Construction, Illumina Sequencing, Assembly, and Annotation

The sequencing library was prepared following the standard instructions of cDNA preparation using the Illumina HiSeq X-ten platform. The raw sequencing reads were first processed to eliminate adapter sequences and low-quality bases using Trimmomatic v0.38 [45], with default parameters. Next, bases with a quality score below Q30 were trimmed from both the 3′ and 5′ ends using a sliding window of 5 bp, and reads shorter than 25 bp were discarded. The quality of the processed sequences was evaluated with FastQC (version 0.11.5). De novo assembly of the filtered reads was performed using Trinity (version 2.0.2, https://github.com/trinityrnaseq/trinityrnaseq/releases, accessed on 20 September 2021), and assembly statistics were generated via the Trinity_stats.pl script [46]. The coding sequence (CDS) was identified using Transdecoder (version 5.5.0) [47]. The completeness of transcriptome assembly was assessed using Benchmarking Universal Single-Copy Orthologs (BUSCO, version 5.2.2) with default parameters, referencing conserved orthologs specific to insects (https://busco.ezlab.org/, accessed on 18 March 2021) [48].

All transcripts were searched against the National Center for Biotechnology Information (NCBI) non-redundant protein sequences database (NR) using the Basic Local Alignment Search Tool (BLAST, E-value < 10^−5^, https://blast.ncbi.nlm.nih.gov, accessed on 18 October 2021); the top-hit transcripts were selected as unigenes. The sequence direction and amino acid sequence prediction for unigenes that could not be aligned to any terms were estimated using Transdecoder [46] in the Trinity program. To annotate unigenes, sequences were searched in GO (Gene Ontology) [49], KEGG [50] (Kyoto Encyclopedia of Genes and Genomes), COG (Clusters of Orthologous Groups), and Pfam (Protein Family), using the EggNOG (Non-supervised Orthologous Groups) database (http://eggnog.embl.de/, accessed on 18 July 2021) [51], which is a database of orthologous groups of genes.

### 2.3. Ortholog Identification and Phylogenetic Analysis

The orthologous relationships of different ant species were inferred using OrthoFinder software (version 2.2.6) [52]. To visualize the intersections between groups, the R package UpSetR [53] was selected due to the high number of intersections. We aligned and computed the phylogenetic trees for the orthogroups. The maximum likelihood (ML) method was used to reconstruct the species tree with IQ-TREE version 1.6.8 [54]. The branch support and single branch test values were estimated using the embedded ultrafast bootstrap approach (UFBoot) [55].

### 2.4. Index of Codon Usage Bias

Codon usage bias is typically assessed using three principal indices: the relative synonymous codon usage (RSCU), the codon adaptation index (CAI), and the effective number of codons (ENC) [56,57]. This bias is shaped by various factors, such as GC content, mutational pressures, natural selection, levels of gene expression, and the length of encoded proteins. Due to evolutionary constraints, closely related species often display comparable patterns of codon usage [58].

In this research, we utilized CodonW version 1.4.2 to compute three key indicators of codon usage bias: the ENC, the CAI, and the RSCU. The ENC metric reflects the extent of codon preference, where values span from 20 (indicating extreme bias) to 61 (suggesting no bias); lower ENC scores denote a stronger inclination toward certain codons [59]. The CAI provides insight into codon preferences in highly expressed genes, with values ranging from 0 to 1; higher scores typically correlate with greater expression efficiency and a stronger bias toward optimal codons [60]. Meanwhile, RSCU quantifies the usage frequency of synonymous codons relative to uniform usage and is calculated as follows:

$$RSCU_{ij}=\frac{X_{ij}}{\frac{1}{N_{i}}\sum_{j=1}^{N_{i}}X_{ij}}$$

where X_ij_ represents the frequency of the j-th codon encoding the i-th amino acid, and N_i_ denotes the total number of codons encoding the i-th amino acid [61]. A codon with an RSCU value exceeding 1 is considered to be used more frequently than expected among its synonymous codons [18,62]. To illustrate codon usage trends, a heatmap was constructed using R, where codons were organized based on their nucleotide composition: the first and second nucleotide positions were mapped to the y-axis, while the third nucleotide position was displayed along the x-axis.

### 2.5. Factors Influencing Codon Usage Bias

To assess the role of mutational pressure in codon usage bias, we utilized a neutrality plot. This plot displays the GC content at the third codon position (GC3) on the x-axis and the average GC content of the first and second positions (GC12) on the y-axis. A high positive correlation between GC3 and GC12, with a correlation coefficient near 1, indicates that mutation pressure predominantly governs codon usage, while natural selection exerts a minimal effect [63].

Additionally, ENC-GC3 plots were generated to examine the effects of base composition and selection pressure on codon usage. In these plots, ENC values are plotted against GC3 values, and the expected ENC values are calculated using the following equation:

$$ENC_{exp}=2+GC3_{s}+\frac{29}{GC3_{s}^{2}+{\left(1-GC3_{s}\right)}^{2}}$$

The standard ENC curve represents the theoretical codon usage expected under neutral conditions, based solely on GC3 content. When gene data points align closely with or lie along this curve, it suggests that codon usage bias is primarily shaped by mutational pressure acting on the third codon position. By contrast, genes that fall significantly below the curve are indicative of additional influences, particularly natural selection [64,65]. Previous studies have demonstrated that a close fit to the expected ENC curve implies that GC3 composition largely governs codon usage patterns, whereas marked deviations beneath the curve highlight selective constraints as the prevailing factor [64].

To gain deeper insights into base composition bias at the third codon position, we examined the synonymous codon base distributions across eight Formicidae species. Two compositional ratios—G3/(G3 + C3) and A3/(A3 + T3)—were plotted along the x-axis and y-axis, respectively. If codon usage bias was solely the result of mutational pressure, one would expect an approximately equal distribution of base frequencies at the third codon position, with A ≈ T and G ≈ C. Any significant departure from this expectation is indicative of selective influence on codon choice.

Additionally, to assess the relationship between gene expression and codon usage bias, a scatter plot was constructed using CAI values on the x-axis and ENC values on the y-axis [66,67]. This visualization enables evaluation of how codon bias correlates with expression levels across genes.

### 2.6. Functional Enrichment

Among the eight desert species, six of them are native to the Mu Us desert. The other two species (*C. aenescens* and *T. tsushimae*) colonized this environment after artificial planting or aerial sowing of herbs and shrubs. In order to compare the metabolism of native desert ants with species introduced as a result of habitat restoration, we enriched the fourth level orthogroup gene functions using GO enrichment analysis. We focused specifically on the biological process (BP) category and selected the top eight significantly enriched GO terms (FDR-adjusted *p* < 0.05) that were functionally relevant to metabolic activity, stress response, and environmental adaptation. These terms were prioritized for their potential ecological relevance in distinguishing native species from those introduced via habitat restoration.

## 3. Results

### 3.1. Transcriptome Assembly, Quality Assessment, and Annotation

**Figure 1:**
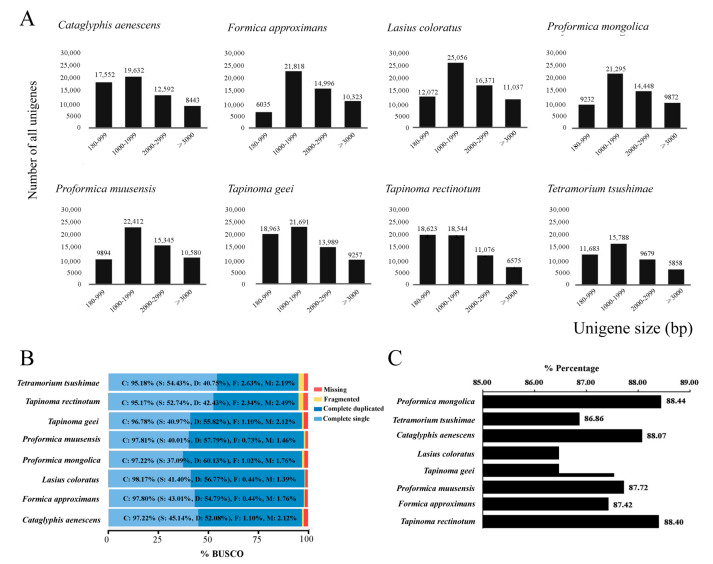
(**A**) The length distributions of the the assembled unigenes of eight transcriptome sequences. The x-axis shows sequence length of the unigenes and y-axis is the number of unigenes. (**B**) Quality assessment of optimised transcriptomes. Percentages of BUSCOs identified when searched against the insect orthologs (Insecta_odb10). (**C**) Percentages of protein functional annotation of eight RNA sequences.

To optimize transcript identification, high-quality RNA samples were extracted from four to seven worker ants of each species and then pooled in equal amounts for library preparation and sequencing. The transcriptome was assembled using Trinity software (version 2.5.1). A summary of the transcriptomes is presented in Table 1. *T. tsushimae* had the lowest number of transcripts at 52,223, and *L. coloratus* had the highest number of transcripts at 78,659. Averages of 67,755 transcripts and 56,341 unigenes were obtained. *P. muusensis* had the highest N50 value at 4531 bp, and *T. rectinotum* had the lowest value at 3035 bp, with an overall average N50 value of 3959 bp. The distribution of all unigenes with different lengths is shown in Figure 1A. All assembled unigenes exceeded 180 bp in length, with approximately 40% being longer than 2000 bp on average. In seven of the eight transcriptomes, the majority of unigenes ranged from 1000 to 1999 bp, while *T. rectinotum* showed a different distribution pattern. The BUSCO of eight transcriptomes was run with default parameters on a set of 1367 conserved insect orthologs (Insecta_odb10), which were found to be >95% complete (an average of 96.9%, single: 44.3%, duplicated: 52.6%), 1.2% fragmented, and 1.9% missing (Figure 1B). BUSCO analysis indicated a comparatively high degree of assembly and annotation completeness [68]. The RNA-seq library consisted of an average of 31,670 protein-coding genes, of which 88% were annotated (Figure 1C). These transcripts had hits against the EggNOG database, including the GO, KEGG, Pfam, and COG databases (Figure S1).

### 3.2. Orthogroup Identification and Phylogenetic Analysis

**Figure 2:**
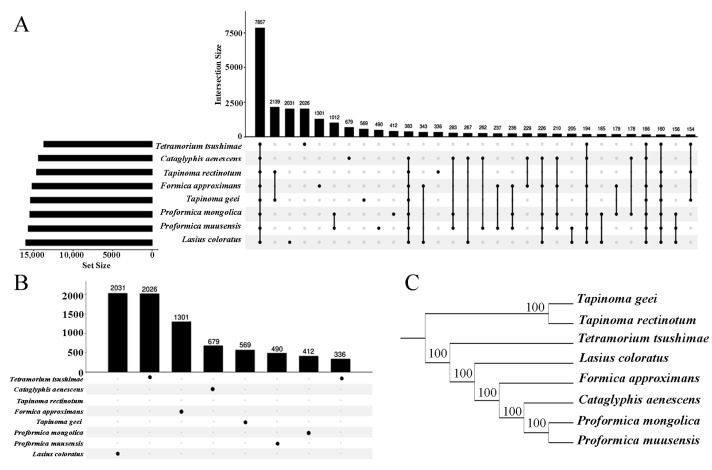
(**A**) Distribution of gene counts assigned to orthogroups across eight species. (**B**) Number of species-specific orthogroups per species. Numbers above bars indicate the number of orthogroups shared at each intersection. (**C**) Phylogenetic relationships among the eight species inferred from OrthoFinder analysis.

The gene orthologous analysis identified 7857 orthogroups representing the eight ant species (Figure 2A), and a fraction of the genes (1.3–3.7%) belonged to species-specific groups (Figure 2B). *L. coloratus* had the most species-specific genes (2031), and *T. rectinotum* had the least species-specific genes. Between 93.9% and 96.3% of the genes across the eight species were assigned to orthogroups (Table S1). A total of 175 single-copy orthogroups, i.e., groups containing exactly 1:1 ortholog proteins, were identified. Robust phylogenetic trees were inferred in IQ-TREE from 175 single-copy genes, using best-fit amino acid and codon models and ultrafast bootstrap support (UFboot = 100). As expected, the unrooted tree showed that *T. geei* was a sister group to *T. rectinotum*, and *P. mongolica* was more closely related to *P. muusensis*, followed by *C. aenescens* (Figure 2C). *Proformica* and *Cataglyphis* were nested in *Formica*. The phylogenetic relationships between the genera were consistent with the ML analysis of a previous study [69].

### 3.3. Nucleotide Composition and Codon Positions in CDSs

We quantified the GC contents at the first, second, and third codon positions (GC1, GC2, and GC3), as well as the overall GC content (GCall), for coding sequences (CDSs) in the transcriptomes of eight ant species (Table 2). An asymmetrical distribution of nucleotides was observed at the third codon position across all species. In these Formicidae species, GC1 ranged from 48.84% to 50.09%, GC2 ranged from 38.87% to 39.76%, and GC3 ranged from 43.11% to 48.91%. The GC content at each codon position was generally below 50%, indicating a preference for codons ending in A or T. Overall, the CDS base compositions and codon usage patterns were highly consistent among the eight species.

**Figure 3:**
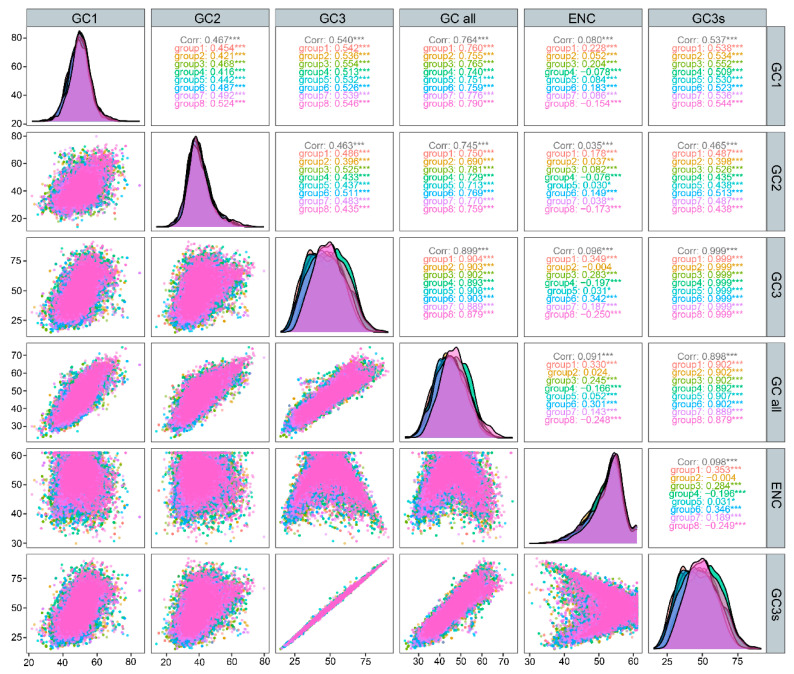
Correlation analysis of GC content at different codon positions. *p* < 0.05 indicates a statistically significant correlation (*),and *p* < 0.001 reflects a very highly significant correlation (***). Ant species are color-coded consistently across the upper-right labels, the middle density plots, and the lower-left scatter plots (group 1: *P. mongolica*; group 2: *T. geei*; group 3: *C. aenescens*; group 4: *L. coloratus*; group 5: *T. rectinotum*; group 6: *P. muusensis;* group 7: *F. approximans*; and group 8: *T. tsushimae*).

GC content is a key indicator of genomic composition. To further explore codon usage bias, we examined the correlations among GC1, GC2, GC3, and the overall GC content (GCall) (Figure 3). GCall showed a significant positive correlation with GC1, GC2, and GC3 (*p* < 0.001). In addition, GC1 and GC2 were also significantly correlated with each other (*p* < 0.001).

### 3.4. Analysis of Codon Usage Indicator

To evaluate codon usage bias across the eight Formicidae ant transcriptomes, we analyzed the CAI and the ENC for all predicted coding sequences (CDSs). The CAI values ranged from 0.198 to 0.208, indicating low overall gene expression levels and suggesting weak codon preference across the transcriptomes (Table 3). CAI values closer to 1 generally reflect higher gene expression and stronger codon usage bias.

The ENC values were used to further assess codon usage patterns. In general, ENC < 35 reflects strong codon bias, ENC values between 35 and 50 indicate moderate or weak bias, and ENC > 50 suggests very weak or nearly random codon usage. As shown in Table 3, the average ENC value across the eight species was approximately 56.17, supporting the conclusion of weak codon usage bias in Formicidae ants.

**Figure 4:**
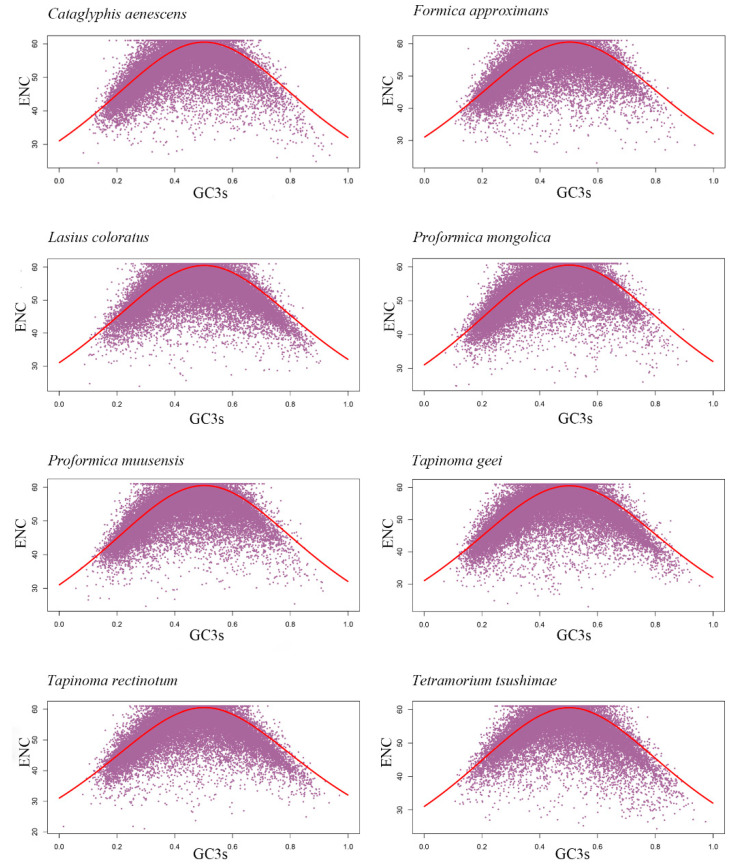
ENC plots showing the relationships between GC3 content (x-axis) and ENC values (y-axis) for transcriptome CDSs of eight ant species.

We next examined the ENC-GC3 plot to determine the influence of mutational bias versus natural selection. In this plot, genes located on or near the standard curve are assumed to be primarily shaped by mutational bias, while genes deviating significantly from the curve may be subject to natural selection or other evolutionary forces [70]. Our results showed that only 21% to 23% of genes per species were located on or near the standard curve (Figure 4; Table 4), suggesting that most genes were influenced by selective pressures beyond mutational bias.

We further quantified the overall strength of codon bias using ENC thresholds. On average, only 196 genes (0.62%) per species had ENC values below 35, indicating strong codon bias. By contrast, the vast majority of genes (approximately 15,730 per species) had ENC values greater than 35. These included an average of 11,321 genes (35.76%) with moderate codon bias (ENC 35–50) and 20,140 genes (63.62%) with very weak bias (ENC > 50).

To more accurately assess the extent to which the observed ENC values diverged from expected values, we computed the relative deviation using 5000 permutations of the ratio (ENCexp−ENCobs)/ENCexp for each gene. The most frequently observed deviation ratios ranged from 0.03 to 0.09, while values between 0.05 and 0.07 were less common (Figure S2). These results indicated that, in most cases, the observed ENC values were lower than expected, supporting the influence of selection pressures on codon usage in many genes.

Overall, both the CAI and ENC analyses consistently indicate a weak but detectable codon usage bias in the eight Formicidae ant species studied.

### 3.5. PR2 Plot Analysis of Eight Species

**Figure 5:**
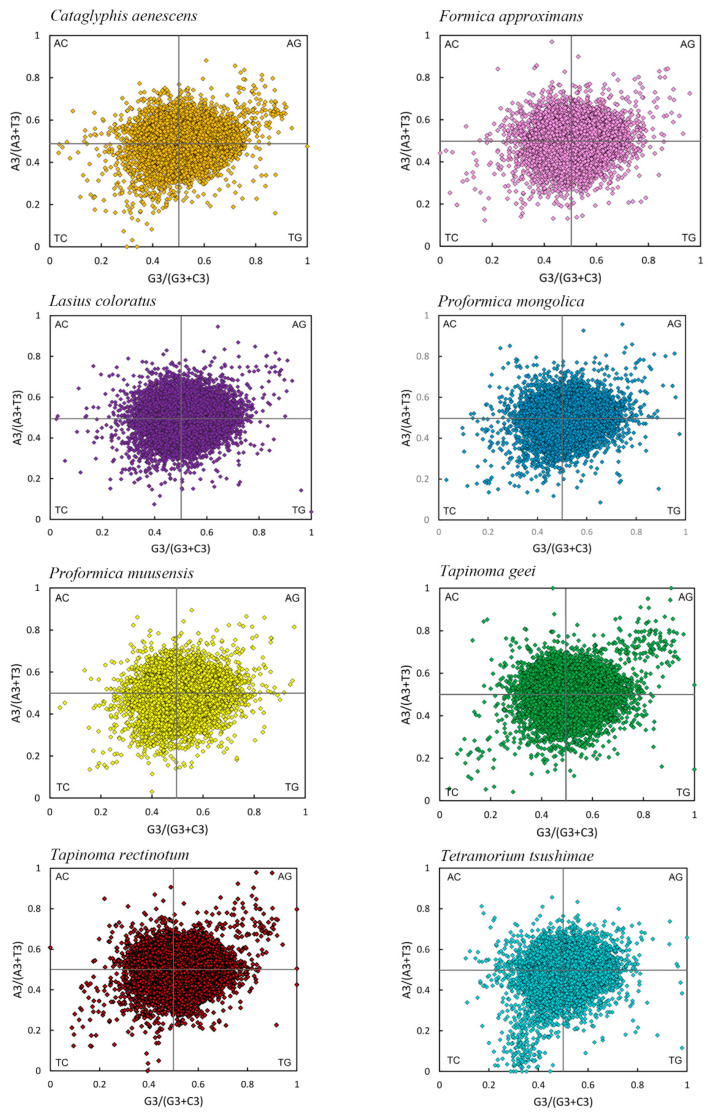
PR2 plots of the transcriptome CDSs of eight species.

PR2 plots were used to investigate the relative influence of mutation and natural selection on codon usage bias (CUB) by analyzing the balance between A/T and G/C at the third codon position. As shown in Figure 5, the distribution of genes across the four quadrants was not entirely symmetrical, suggesting some degree of compositional asymmetry. However, when examined along the vertical axis (A vs. T) and horizontal axis (G vs. C), most genes in all eight species were broadly scattered on both sides of the midlines, without strong clustering toward either base. This pattern indicated no substantial overall bias toward A over T or G over C at the third codon position in most species.

For instance, in *T. rectinotum*, *T. geei*, *P. muusensis*, *L. coloratus*, *C. aenescens*, *F. approximans*, and *P. mongolica*, genes were approximately balanced between the upper right (Quadrant I) and lower left (Quadrant III), supporting the conclusion of minimal nucleotide bias. By contrast, *T. tsushimae* exhibited a noticeable concentration of genes in Quadrant III, suggesting a relative preference for T and C at the third codon position (Figure 5).

### 3.6. Differential Analysis of Synonymous Codon Usage

**Figure 6:**
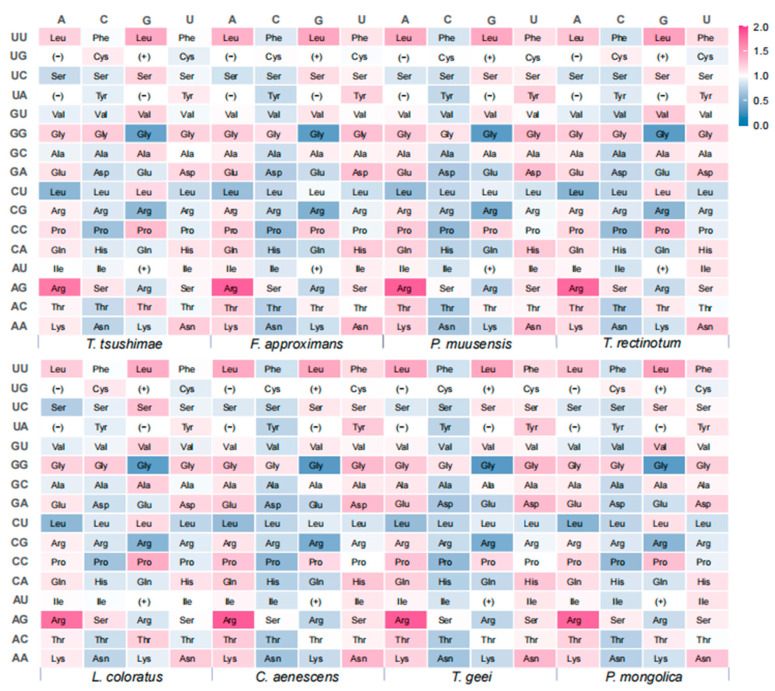
Heat map of relative synonymous codon usage (RSCU) values of eight ant species. The vertical axis represents the first two bases of the codons, and the horizontal axis represents the third base corresponding to each species. (−) indicates stop codons (UAA, UAG, and UGA). (+) indicates nonsynonymous codons (AUG and UGG).

For the differential analysis, three stop codons (UAA, UAG, and UGA) and two non-synonymous codons (AUG and UGG) were excluded, resulting in 59 synonymous codons being included in the analysis (Figure 6). Among these, the codon AGA (Arg) showed the highest relative synonymous codon usage (RSCU) value, followed by UUG (Leu). Overall, the codon usage patterns were consistent across the eight ant species, with each species exhibiting 27 to 33 preferred synonymous codons (RSCU > 1), most of which ended in A or U. Codons ending in G were less commonly preferred, and those ending in C were the least favored. In particular, *F. approximans*, *P. muusensis*, *C. aenescens*, and *T. geei* each showed a preference for 33 codons ending in A or U. *T. rectinotum* and *P. muusensis* preferred 32 such codons, while *T. tsushimae* and *L. coloratus* showed the fewest, with 29 and 27, respectively. These results suggested a shared codon usage bias across species in the family Formicidae, with a notable preference for A/U at the third codon position.

### 3.7. Neutrality Plot Analysis of Eight Species

**Figure 7:**
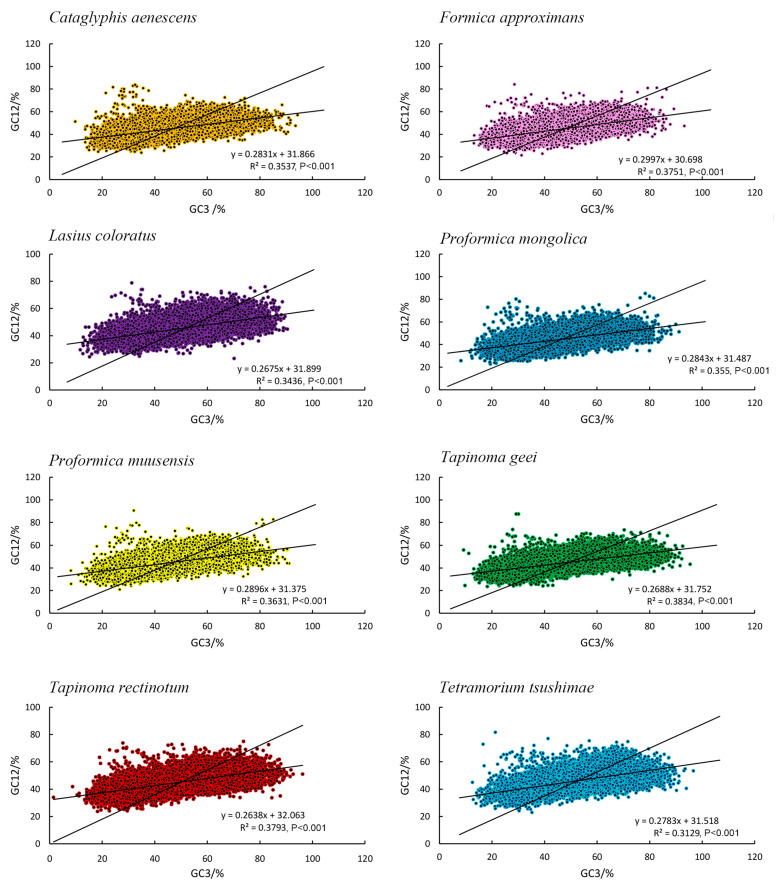
Neutrality plots of the transcriptome CDSs of eight species.

The correlation between GC12 and GC3 was low in all ant species, with coefficients ranging from 0.2638 to 0.3834, indicating only a weakly positive relationship between these codon positions. This weak correlation suggested that the evolutionary forces acting on the first and second codon positions (GC12) differed from those on the third position (GC3). This suggested that both mutational pressures and natural selection influenced codon usage in these species, although natural selection may have had a more significant impact on shaping codon preferences, as observed in other studies (Figure 7). This result was in agreement with the ENC plot and PR2 plot analyses.

### 3.8. GO Enrichment of Ants Occupying Different Desert Niches

**Figure 8:**
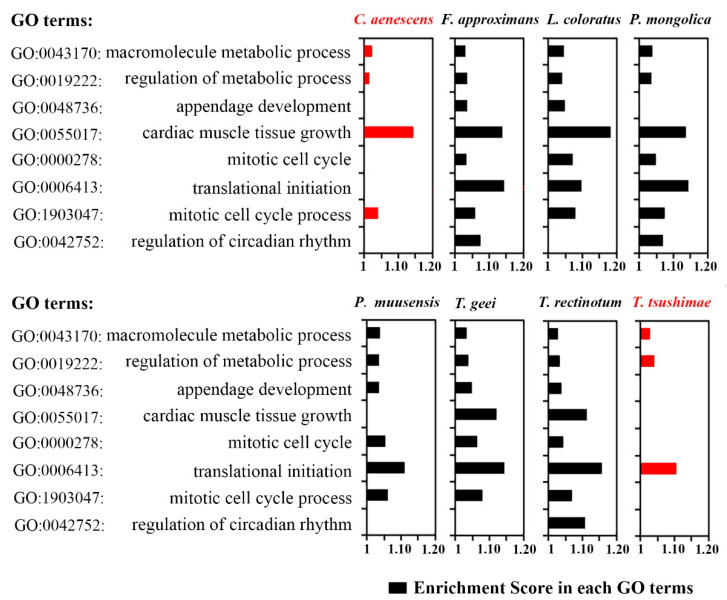
Enrichment scores of physiological activity-related genes by GO term. The red bars represent ants in artificially planted desert areas; the black bars represent native desert ants.

Owing to specific physiological adaptations, desert ants have an unusually high thermal tolerance and can stay active even during the hottest periods of the day [71]. In desert ants, physiological adaptations are most likely to occur in metabolic activity. We screened eight GO terms from the results of the GO enrichment analyses: GO: 0042752, GO: 1903047, GO: 0006413, GO: 0000278, GO: 0055017, GO: 0048736, GO: 0019222, and GO: 0043170. These genes were associated with certain metabolic parameters, including “mitotic cell cycle process,” “mitotic cell cycle,” “regulation of metabolic process,” and “macromolecule metabolic process” (Figure 8). Compared with ant species in the desert, the two ant species occupying the artificially planted areas had significantly lower enrichment scores related to metabolic activity, and a few genes were not enriched at all (red bars in Figure 8). These findings suggested that the native ants expressed more genes related to metabolism, which contributed to their proliferation in this extreme environment.

## 4. Discussion

Codon usage bias (CUB) is a common characteristic across both prokaryotic and eukaryotic genomes. Two primary frameworks have been proposed to explain this pattern: the selection–mutation–drift balance model and the neutral theory. The former suggests that codon usage arises from the combined influence of mutation bias, random genetic drift, and widespread but weak natural selection. By contrast, the neutral theory considers synonymous codon variation largely as a result of stochastic events, assuming little to no selective constraint at these sites.

Nucleotide composition, particularly GC content, is widely acknowledged as a major determinant of codon usage patterns, often reflecting underlying mutational biases. Due to differences in mutational bias, GC content can vary substantially among species, even within the same taxonomic order. Previous studies have shown that genes in hymenopteran genomes are typically located in GC-poor regions, the same as our result, whereas such compositional bias is less apparent in dipteran genomes [23].

Codon usage bias shows substantial variation across different lineages, not only in its overall strength but also in the direction of codon preference. For instance, the genomes of parasitoid wasps are typically AT-rich, a feature that aligns with their tendency to favor codons ending in A or T [26]. Conversely, Drosophila melanogaster exhibits a higher GC content in its coding sequences and displays a strong preference for GC-ending codons [24]. In our analysis, we found that ants, like butterflies and honeybees, also show a pronounced bias toward AT-ending codons [72,73,74,75]. These observations support the idea that codon usage in ants may, at least partly, be driven by underlying genomic base composition, as has been suggested for various other organisms including prokaryotes, plants, humans, and flatworms [76,77,78]. In our study, despite occupying diverse ecological habitats, the ant species showed no substantial variation in either nucleotide composition or codon usage bias. Due to the AT-rich and GC-poor composition of transcriptome CDSs across all eight ant species, codons ending in A or T were utilized more frequently—and with similar frequencies—than those ending in G or C. This pattern supports the notion that mutation pressure, rather than translational selection, is the primary driver shaping synonymous codon usage in these ant species.

Codon usage bias is commonly assessed using the Effective Number of Codons (ENC) metric. An ENC value of 61 reflects no codon preference, whereas a value of 20 indicates extreme bias, where only one codon is used per amino acid. In our study, over 99% of genes exhibited ENC values above 35, and approximately 60% surpassed an ENC of 50. These results indicate that codon usage bias was generally weak across transcriptome coding sequences in Formicidae species. Our analysis of codon bias and nucleotide composition bias was based on comparison between CAI and ENC. As CAI is a directional measure of codon bias unlike ENC, the CAI of genes dominated by mutation bias would have a lower value than those with completely even usage of codons. Whether ENC or CAI, the results of the eight ant species were similar, indicating a high degree of similarity in the regulation of their expression [79].

In our study of eight ant species, although overall codon usage patterns were broadly similar among the eight desert ant species, notable interspecies differences were detected. For example, *L. coloratus* exhibited a slightly higher GC content at the third codon position (GC3) compared to species like *P. mongolica* and *C. aenescens*, which maintained stronger AT-rich codon preferences. In this study, *T. geei* and *T. rectinotum* exhibited the highest proportion of CDSs with ENC values less than 35 among the eight desert ant species analyzed. Since lower ENC values generally indicate a stronger codon usage bias, this finding suggests that these two species are subject to stronger codon usage selection. Considering their close phylogenetic relationship, it can be inferred that *T. geei* and *T. rectinotum*, belonging to the same genus, share similar genetic backgrounds that may have contributed to their pronounced codon bias. Therefore, phylogenetic relationships appear to have a greater impact than environmental factors in shaping their codon usage patterns. Similarly, *P. mongolica* and *P. muusensis* exhibited only modest shifts in codon usage patterns, as revealed by the PR2 plot analysis, further supporting the notion that phylogenetic constraints play a more significant role than environmental pressures in determining codon usage bias among closely related species.

The GO enrichment analysis revealed that desert ants exhibit significant upregulation of genes involved in metabolic processes, particularly those related to the mitotic cell cycle and macromolecule metabolism. These differences suggest that metabolic regulation is a key physiological adaptation to the extreme desert environment. Thus, although codon usage bias is primarily determined by phylogenetic history, gene expression changes play an essential role in environmental adaptation.

In summary, our findings demonstrate that while phylogenetic history predominantly determines codon usage bias, environmental factors drive differential gene expression to meet specific physiological demands. This dual influence sheds light on the adaptive strategies employed by desert ants in extreme environments.

## 5. Conclusions

In this study, we conducted a codon usage bias analysis of genes from eight ant species using transcriptome data. The GC content of coding sequences (CDSs) ranged from 43.61% to 46.2%, indicating a slightly AT-rich nucleotide composition. Analysis of optimal codons revealed a general preference for A- or U-ending synonymous codons across all eight *Formicidae* species. These results suggest that nucleotide composition plays a major role in shaping codon usage bias in these ants. Moreover, codon usage bias was also influenced by gene expression level. A total of 27 to 33 optimal codons were identified in each species, most of which ended in A or U.

Our analysis helps to illuminate regular evolutionary patterns, identify novel genes, and optimize heterologous expression systems through the overall codon usage bias analysis. We also found that phylogeny plays a more significant role than environmental pressures in determining codon usage bias among closely related species. Overall, these findings provide new insights into codon usage patterns in Formicidae and establish a valuable foundation for future gene engineering and functional genomics studies in ants.

## Figures and Tables

**Table 1:** Summary of assembly statistics for eight transcriptome sequences.

	*C. aenescens*	*F. approximans*	*L. coloratus*	*P. mongolica*	*P. muusensis*	*T. geei*	*T. rectinotum*	*T. tsushimae*
Row reads (bp)	6,811,039,804	8,326,823,594	7,065,179,209	7,174,050,294	8,181,571,346	7,158,103,337	7,552,263,822	7,217,089,193
GC%	39.94	41.08	43.14	38.95	40.22	40.56	41.45	40.66
**Assembly**								
Trinity Transcripts (n)	68,389	64,382	78,659	66,227	70,312	77,719	64,130	52,223
Total unigenes (n)	58,219	53,172	64,536	54,846	58,231	63,900	54,818	43,008
Maximum length (bp)	35,719	29,880	33,377	28,270	29,773	27,646	24,454	18,282
Minimum length (bp)	189	183	186	197	189	184	182	201
N50	4052	4450	4183	4214	4531	3775	3035	3430
GC%	38.62	39.01	40.64	37.90	38.59	39.46	39.80	41.16
**BUSCO (%)**								
Complete	97.22	97.80	98.17	97.22	97.81	96.78	95.17	95.18
Fragmented	1.10	0.44	0.44	1.02	0.73	1.10	2.34	2.63
Missing	1.68	1.76	1.39	1.76	1.46	2.12	2.49	2.19

**Table 2:** Summary table of eight ant species.

Species	CBI	FOP	L_aa	GC1%	GC2%	GC3%	GCall%
*P. mongolica*	−0.038	0.398	381	48.84	38.87	43.11	43.61
*T. geei*	−0.019	0.409	368	49.19	38.89	45.98	44.69
*C. aenescens*	−0.036	0.399	397	49.13	39.28	43.18	43.86
*L. coloratus*	−0.005	0.416	403	49.94	39.76	48.91	46.2
*T. rectinotum*	−0.014	0.412	361	49.4	39.01	46.34	44.91
*P. muusensis*	−0.033	0.4	398	49.03	39.14	43.8	43.99
*F. approximans*	−0.031	0.402	408	49.05	39.1	44.59	44.25
*T. tsushimae*	0.005	0.422	364	50.09	39.58	48.46	46.04

**Table 3:** Summary table of ENC of eight ant species.

Species	ENC	CAI	ENC < 35	35 ≤ ENC ≤ 50	50 < ENC
*P. mongolica*	55.71	0.198	169	11,926	20,927
*T. geei*	56.28	0.204	266	13,152	20,288
*C. aenescens*	55.74	0.198	156	11,235	18,543
*L. coloratus*	56.52	0.202	161	12,124	23,836
*T. rectinotum*	56.31	0.205	259	10,948	16,751
*P. muusensis*	55.93	0.198	160	12,360	22,237
*F. approximans*	56.13	0.198	148	11,518	21,741
*T. tsushimae*	56.74	0.208	247	7308	16,795

**Table 4:** Frequency distribution of ENC ratio of eight ant species.

Species Name		[−0.25, −0.15)	[−0.15, −0.05)	[−0.05, 0.05)	[0.05, 0.15)	[0.15, 0.25)	[0.25, 0.35]
*P. mongolica*	Frequency	4352	20,241	7093	368	12	1
	Frequencies	0.14	0.63	0.22	0.01	0.00	0.00
*T. geei*	Frequency	4949	20,297	6874	395	14	1
	Frequencies	0.15	0.62	0.21	0.01	0.00	0.00
*C. aenescens*	Frequency	3967	18,379	6202	312	18	1
	Frequencies	0.14	0.64	0.21	0.01	0.00	0.00
*L. coloratus*	Frequency	4862	22,432	7246	387	4	2
	Frequencies	0.14	0.64	0.21	0.01	0.00	0.00
*T. rectinotum*	Frequency	4112	16,792	5753	301	9	1
	Frequencies	0.15	0.62	0.21	0.01	0.00	0.00
*P. muusensis*	Frequency	4541	21,222	7519	348	15	0
	Frequencies	0.13	0.63	0.22	0.01	0.00	0.00
*F. approximans*	Frequency	4166	20,643	7115	316	10	0
	Frequencies	0.13	0.64	0.22	0.01	0.00	0.00
*T. tsushimae*	Frequency	3312	14,355	5414	261	11	0
	Frequencies	0.14	0.61	0.23	0.01	0.00	0.00

## Data Availability

The following information is supplied regarding the availability of RNA sequences: the raw sequence data are deposited in CNCB’s GSA under accession number CRA024798.

## Supplementary Materials

The following supporting information can be downloaded at: https://www.mdpi.com/article/10.3390/genes16070749/s1, Figure S1. Annotation results of eight transcriptomes; Figure S2. Frequency distribution of ENC in eight ant species; Table S1. Per species general statistics of KEGG result; Table S2. Per species general statistics of the OrthoFinder analysis showing the orthogroup size and relative proportion of genes assigned to orthogroups.
