# Supplementary material for: Comparative Analysis of Codon Usage Bias in Transcriptomes of Eight Species of Formicidae
Source: Genes (Basel). 2025 Jun 27;16(7):749. doi: 10.3390/genes16070749 (PMC12294360; doi:10.3390/genes16070749)
Supplement: Supplementary file 1 [file genes-16-00749-s001.zip › genes-3660498-supplementary.pdf]

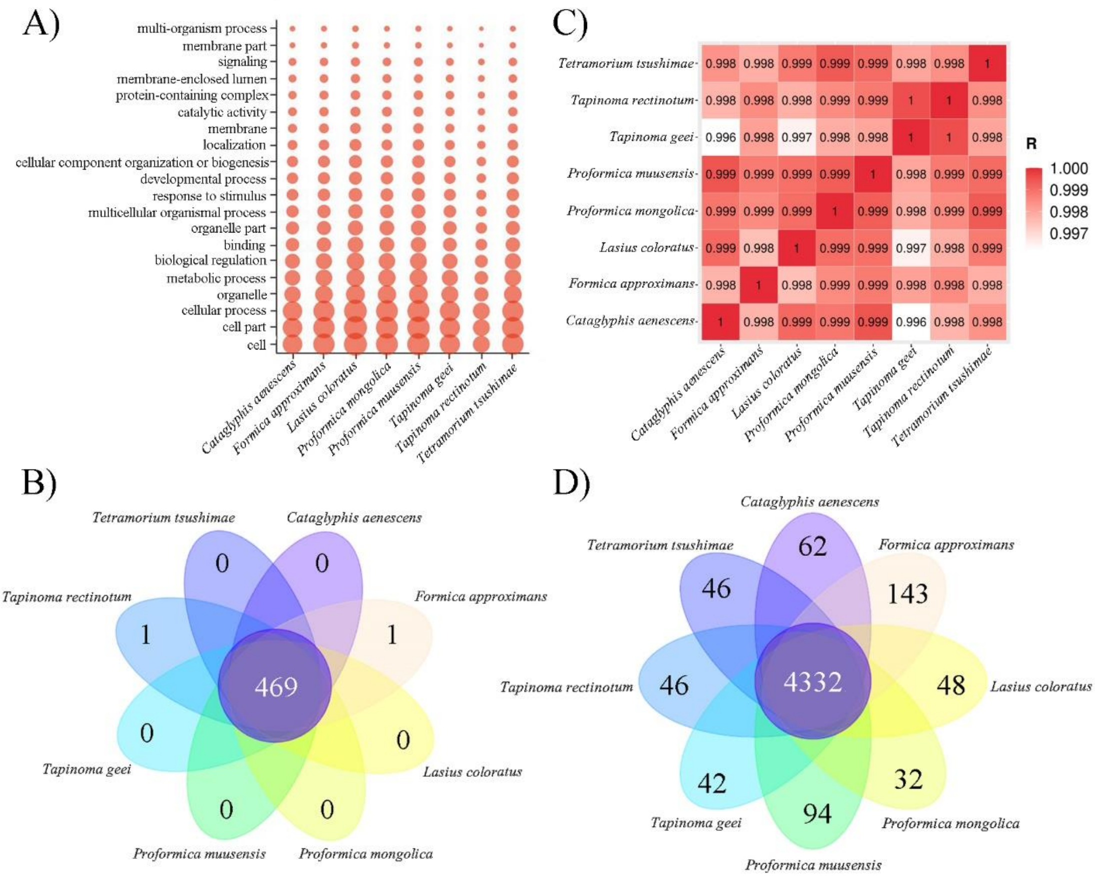

Figure S1. Annotation results of eight transcriptomes: (A) The top 20 of GO annotation in eight transcriptomes; (B) Venn diagram to show the number of shared pathways and number of pathways unique to each species by KEGG database; (C) Correlation matrix showing relationship between all samples as well as replicates; (D) Venn diagram to show the number of shared genes and number of genes unique to each species by Pfam database.

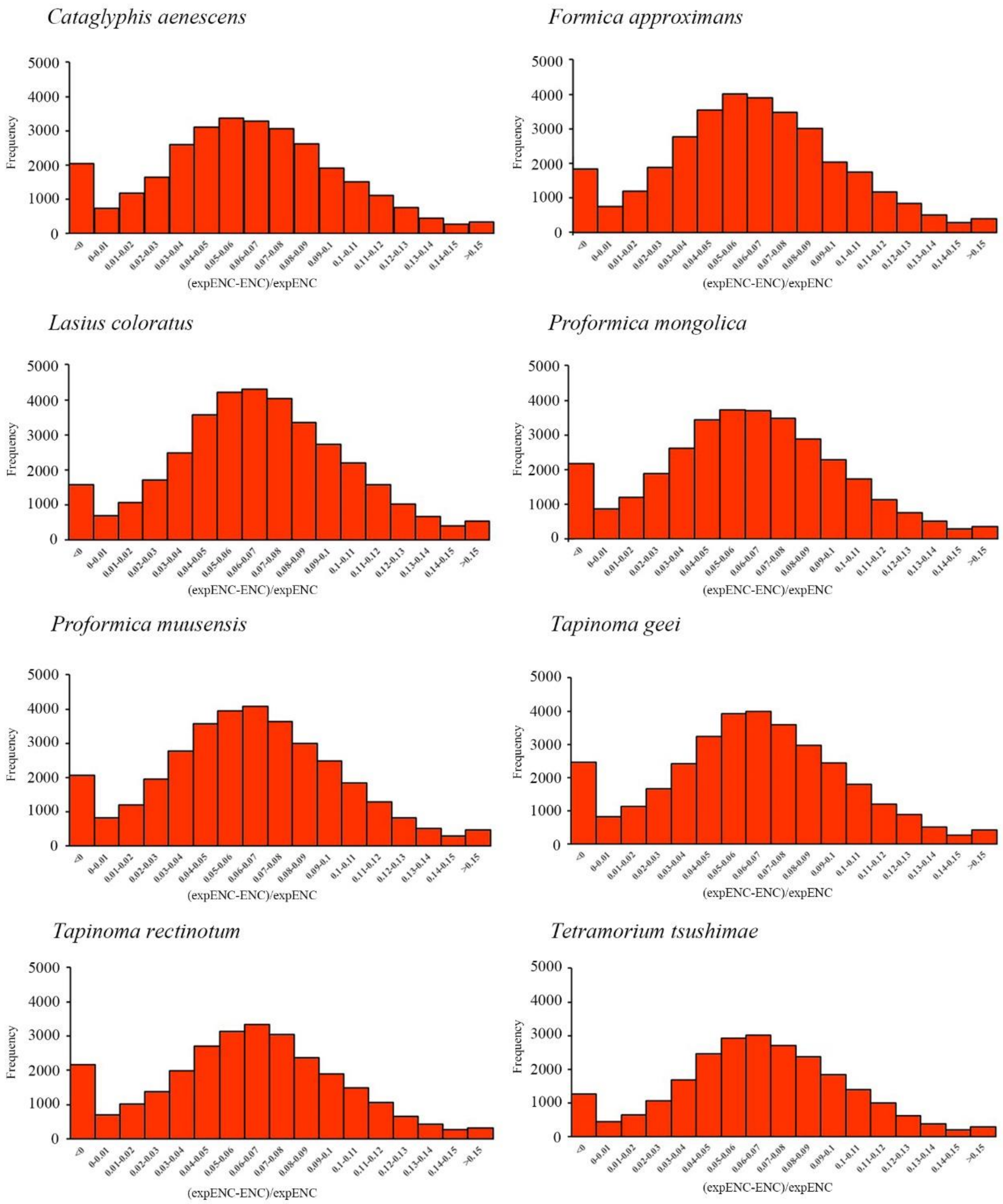

Figure S2. Frequency distribution of ENC in eight ant species.

Table S1 Per species general statistics of KEGG result

	Cataglyphis aenescens	*Formica approximans*	*Lasius coloratus*	*Proformica mongolica*	*Proformica muusensis*	*Tapinoma geei*	*Tapinoma rectinotum*	*Tetramorium tsushimae*
transcripts	10516	8426	12320	10761	11939	10761	8969	11294
pathways	1615	1608	1592	1575	1627	1575	1571	1592

Table S2 Per species general statistics of the OrthoFinder analysis showing the orthogroup size and relative proportion of genes assigned to orthogroups.

	Cataglyphis aenescens	*Formica approximans*	*Lasius coloratus*	*Proformica mongolica*	*Proformica muusensis*	*Tapinoma geei*	*Tapinoma rectinotum*	*Tetramoriu m tsushimae*
Number of genes	29950	33422	36131	33031	34764	33725	27972	24366
Number of genes in orthogroups	28134	32027	34065	31806	32964	32071	26519	22908
Number of unassigned genes	1816	1395	2066	1225	1800	1654	1453	1458
Percentage of genes in orthogroups	93.9	95.8	94.3	96.3	94.8	95.1	94.8	94
Percentage of unassigned genes	6.1	4.2	5.7	3.7	5.2	4.9	5.2	6
Number of orthogroups containing species	14402	15198	16001	15484	15688	15396	14631	13700
Percentage of orthogroups containing species	44.5	46.9	49.4	47.8	48.4	47.5	45.2	42.3
Number of species-specific orthogroups	365	273	499	175	230	351	177	171
Number of genes in species-specific orthogroups	982	777	1349	445	606	893	462	412
Percentage of genes in species-specific orthogroups	3.3	2.3	3.7	1.3	1.7	2.6	1.7	1.7
